
JOURNAL INFORMATION
==============================
NLM Title Abbreviation: Innov Surg Sci
Journal ID: Innov Surg Sci
NLM Journal ID: 101708165
Journal ID: iss

Innovative Surgical Sciences

EISSN: 2364-7485
Publisher: De Gruyter

ARTICLE INFORMATION
==============================
PMCID: PMC11005950
DOI: 10.1515/iss-2024-2002
Article ID: iss-2024-2002
Article version: 1
Subjects: Article

Abstracts DGPRÄC

Electronic publication date: 2024 Apr 9
Publication date: 2024 Apr
Volume: 9
Issue: Suppl1
First page: 12
Last page: 17
Copyright: © 2024 by Walter de Gruyter Berlin/Boston
Copyright year: 2024
Copyright holder: De Gruyter
License: This work is licensed under the Creative Commons Attribution 4.0 International License.
License URL: https://creativecommons.org/licenses/by/4.0/

Page count: 6

==============================

Abstracts – DCK 2024 – 141st Congress of the German Society of Surgery – Leipzig, April 24–26 • DOI 10.1515/iss-2024-2002
Innov Surg Sci 2024; 9 (Special Suppl 1): s12–s17

**Necrotizing breast infection of a breastfeeding mother - a case report**
ID: 770

Alexander Dermietzel[1 2 3], Philipp Wiebringhaus[1 2 3], Tobias Hirsch[1 2 3], Maximilian Kückelhaus[1 2 3], Sonja Dahmann[3]

[1]*Department of Plastic, Reconstructive and Aesthetic Surgery, Hand Surgery , Fachklinik Hornheide, Deutschland*, [2]*University Hospital Muenster, Division of Plastic Surgery, Deutschland*, [3]*Institute of Musculoskeletal Medicine, University of Muenster, Department of Plastic and Reconstructive Surgery, Deutschland*

**Background:**
Scarlet fever is a contagious infection mostly affecting young children caused by Streptococcus pyogenes. Symptoms include high temperature, signs of oropharyngeal infection with swollen neck glands and a rash. Streptococcus pyogenes is a Gram-positive bacterium, which can cause scarlet fever, skin infections and necrotizing fasciitis. Necrotizing fasciitis is a rare and potentially fatal infection of the soft tissue. It is categorized on etiology into types I-IV. Cases of necrotizing mastitis are an even rarer type of infection. We hereby report our experience in a case of necrotizing mastitis of a breastfeeding mother.

**Materials and methods:**
A 35-year-old female patient was admitted to the emergency unit of a rural hospital with signs of sepsis, pneumonia and a necrotizing soft tissue infection of her left breast. Broad-spectrum antibiotics were started, a CT- scan was performed and an incision was made periareolary for tissue sampling. The patient was transferred to our department of reconstructive plastic surgery and radical surgical debridement of necrotic tissue was performed. Sepsis treatment was effective and the patient was released from ICU. Further surgical resection focused on sparing breast tissue and effective antibiotic treatment. After two cycles of vacuum assisted closure therapy split skin grafting was performed for skin closure.

**Results:**
Microbiology confirmed Streptococcus pyogenes as the cause of necrotizing breast infection in our patient. Further investigation showed a scarlet fever infection in one of the patients' children she was breastfeeding. Fast and radical resection of necrotic tissue and calculated i.v. antibiotic led to fast control of the infection. Vacuum assisted closure therapy resulted in advanced tissue granulation of the breast and split skin grafting was performed resulting in sufficient tissue coverage while preserving the nipple-areola-complex.

**Conclusion:**
This rare case shows a transmission of scarlet fever causing Streptococcus pyogenes from child to breastfeeding mother. As described a necrotizing mastitis and sepsis was caused with severe tissue necrosis. Necrotizing breast infections should be treated similar to other necrotizing soft tissue infections. A fast and radical debridement with tissue samples and close monitoring of the patients is necessary with secondary reconstruction. Young mothers should be informed about the possibility of soft tissue and breast infection caused by oropharyngeal infections of the child.

Open Access. © 2024 The Author(s), published by De Gruyter. This work is licensed under the Creative Commons Attribution 4.0 International License.

Abstracts – DCK 2024 – 141st Congress of the German Society of Surgery – Leipzig, April 24–26 • DOI 10.1515/iss-2024-2002
Innov Surg Sci 2024; 9 (Special Suppl 1): s12–s17

**Evalutation of a Chitosan based Nerve Cap for Prevention of Neuromas in the Rat Sciatic Nerve Model**
ID: 682

Tess Klemm[1], Martin Aman[1], Maximilian Mayrhofer-Schmid[1], Daniel Schwarz[2], Martin Bendszus[2], Ulrich Kneser[1], Leila Harhaus-Wähner[1], Arne Böcker[1]

[1]*BG Klinik Ludwigshafen, Hand-, Plastische- und Rekonstruktive Chirurgie, Mikrochirurgie, Schwer-brand-verletzten-zentrum, Ludwigshafen am Rhein, Deutschland*, [2]*Universitätsklinikum Heidelberg , Neurologische Klinik: Neuroradiologie, Heidelberg, Deutschland*

**Background:**
Approximately 3% of traumatic injuries involve peripheral nerves, and if left untreated, can result in the formation of neuromas. Subsequently these can manifest symptoms, including neuropathic pain, paresthesia, numbness, and cold intolerance. Ultimately this leads to functional limitations and reduced quality of life for the affected individuals.
Despite the clinical relevance of this issue and the extensive research and clinical testing of numerous strategies to prevent and treat symptomatic neuromas, no single approach has emerged as the established standard method.
This study aims to explore the effectiveness of combining a nerve cap, serving as a mechanical barrier, with chitosan, a biodegradable synthetic material that has gained increasing importance in the context of treating peripheral nerve injuries.

**Materials and methods:**
Using the rat sciatic nerve model, 48 SD-rats were divided into four groups. To replicate the relevant nerve defect, a nerve segment of at least 5mm was excised. In the first group, this was the only intervention (Control). Additional intervention in the other three groups consisted of bipolar coagulation (Coagulation) or the application of a nerve cap secured with three 9-0 ethilon sutures. One cap was a polylactic based cap (Polylactic), while the other utilized the biomaterial chitosan (Chitosan).
Neuroma-growth and cap-degeneration were assessed through MR neurography in week 6 and 12. After 12 weeks the nerve was resected, and a macroscopic examination was conducted to assess perineural adhesion, neuroma-shape, and cap-degradation. Additionally, axonal density, axon diameter and myelin fiber thickness were evaluated histomorphometrically.

**Results:**
Histomorphometric evaluation and quantitative analysis revealed significant differences in the following parameters among the study groups. Axonal density was significantly reduced in the Control-Group when compared to the two groups with capped neuromas (Chitosan 7799 ± 2976, p = 0.0193; Polylactic 7605 ± 2169, p = 0.0029). When assessing axonal diameters, only the Chitosan-Group showed significantly thicker axons in comparison to the Control Group (0.176 ± 0.068, p = 0.0210). Analysis of the myelin fibers revealed that both the Chitosan- (0.104 ± 0.028, p = 0.0019) and the Polylactic-Group (0.082 ± 0.025, p = 0.0046) exhibited a greater myelin fiber thickness compared to the Control Group.
No notable differences were observed in any histomorphometric categories when comparing the three Intervention-Groups with each other. Further, macroscopic evaluation of the perineural adhesion showed no significant difference between any of the study groups.
Atypical neuroma with distal axonal outgrowth were most frequently observed in the Control- and Coagulation-Groups, while the neuromas in both the Chitosan- and Polylactic-Group presented in a more bulbous shape. Furthermore, the Chitosan-Caps displayed no significant signs of degradation, whereas the Polylactic-Caps had collapsed and were brittle during explantation. These findings were further supported by MR-Neurography, which revealed distal neurite outgrowth in both the Control- and Coagulation-Group, as well as the absence of cap degradation in the other two groups.

**Conclusion:**

The implementation of nerve caps as mechanical barriers resulted in the formation of denser, more typical shaped bulbous neuromas, which are associated with reduced neuropathic pain compared to atypical formed neuromas with distal neurite outgrowth and subsequential lower axonal density. Further histomorphometric analysis indicated that the caps had a positive impact on axon diameter and myelin fiber thickness. Consequently, employing a cap as a mechanical barrier shows a beneficial effect on neuroma formation when contrasted with neurotmesis alone, consolidating its potential in neuroma treatment and prevention.
However, no significant differences were observed among the interventions, indicating that the use of chitosan does not provide any additional advantages. Moreover, the absence of significant cap-degradation raises concerns regarding potential long-term issues, i.e. foreign-body reactions, which warrant further investigation in subsequent studies with extended observation periods.

**Unveiling the Potential: Free Serratus Carpaccio Flap as a Viable Option for Hand and Foot Defects**
ID: 400

Jia Wei Tee[1], Amir Bigdeli[1], Benjamin Thomas[1], Florian Falkner[1], Oliver Didzun[1], Felix Vollbach[1], Ulrich Kneser[1], Emre Gazyakan[1]

[1]*BG Klinik Ludwigshafen, Ludwigshafen am Rhein, Deutschland*

**Background:**
Defects of the hand and foot often require an individualized reconstructive approach due to their unique functional and aesthetic characteristics. Transferred tissues should be thin, pliable, and durable, with free fascial flaps meeting these requirements. This study aimed to evaluate the serratus fascia flap and the serratus carpaccio flap, which is a modification of the fascia flap by including a thin muscle layer with the goal to enhance flap resilience and lower morbidity rates.

**Materials and methods:**
Between January 2000 and December 2017, 31 patients underwent microsurgical reconstruction of the hand or foot either by serratus fascia flap (fascia group) or serratus carpaccio flap (carpaccio group). The serratus fascia flaps were mainly harvested between 2000 and 2012, while the serratus carpaccio flaps between 2013 and 2017. Patient records were reviewed retrospectively regarding demographics, complications, overall flap survival, additional revision surgeries, and surgical outcome. Categorical variables were compared using Fisher's exact while continuous data with the Mann–Whitney tests. Logistic regression was used to examine the correlation between comorbidities and postoperative complication rates. Differences were considered significant when $p < 0.05$.

**Results:**
The fascia group consisted of 17 patients and the carpaccio group of 14 patients. The average patient age in the fascia group was 39.2 ± 10.5 years and, 39.1 ± 14.7 years in the carpaccio group, respectively. Overall complication rates were significantly lower in the carpaccio group than in the fascia group (28.6% vs 70.6%, p=0.03). The rates of overall flap survival (85.7% in carpaccio group vs 74.5% in fascia group, p=0.66) and partial flap loss (14.3% in carpaccio group vs 47.1% in fascia group, p=0.07) did not differ significantly among both groups. One postoperative hematoma of the donor-site requiring revision surgery was reported in the carpaccio group (7.1%, p=0.45) while no donor-site morbidity was reported in the fascia group.

**Conclusion:**
Our findings indicate that the serratus carpaccio flap presents a promising alternative to the serratus fascia flap. This modification has proven to be effective in substantially reducing postoperative complications requiring additional surgical interventions. Therefore, the serratus carpaccio flap can be considered a dependable reconstructive option for addressing complex soft tissue defects of the hand and foot, with complication rates that are within an acceptable range.

### Intraoperative Blood Flow Analysis of Free Flaps with Arteriovenous Loops for Autologous Microsurgical Reconstruction

ID: 513

Alexander Geierlehner[1], Ingo Ludolph[1], Werner Lang[2], Ulrich Rother[2], Raymund Horch[1], Andreas Arkudas[1]

[1]*Plastisch und Handchirurgische Klinik, Uniklinikum Erlangen, Erlangen, Deutschland*,
[2]*Gefäßchirurgische Abteilung, Uniklinikum Erlangen, Erlangen, Deutschland*

**Background:**
Arteriovenous (AV) loops help to overcome absent or poor-quality recipient vessels in highly complex microvascular free flap reconstruction cases. There are no studies on blood flow and perfusion patterns. The purpose of this study was to evaluate and compare intraoperative hemodynamic characteristics of AV loops followed by free tissue transfer for thoracic wall and lower extremity reconstruction.

**Materials and methods:**
This prospective clinical study combined Transit-Time Flowmetry and microvascular Indocyanine Green Angiography for the assessment of blood flow volume, arterial vascular resistance and intrinsic transit time at the time of AV loop construction and on the day of free flap transfer.

**Results:**
A total of 11 patients underwent AV loop creation, of whom five required chest wall reconstruction and six required reconstruction of the lower extremities. In seven of these cases, the latissimus dorsi flap and in four cases the vertical rectus abdominis myocutaneous (VRAM) flap was used as free flap. At the time of loop construction the blood flow volume of AV loops was 466 ± 180 ml/min which increased to 698 ± 464 ml/min on the day of free tissue transfer ($p > 0.1$). After free flap anastomosis the blood flow volume significantly decreased to 18.5 ± 8.3 ml/min ($p < 0.001$).

**Conclusion:**
This is the first study to perform intraoperative blood flow and hemodynamic measurements of AV loops followed by free tissue transfer. Our results show hemodynamic differences and contribute to deeper understanding of the properties of AV loops for free flap reconstruction.

**Challenges in Achieving Radicality of Nasal Basal Cell Carcinoma: Contemplating the Adoption of Novel Techniques?**
ID: 623

Karolina Baltrušaitytė[1], Ernest Zacharevskij[2], Loreta Pilipaityte[2]

[1]*Litauische Universität für Gesundheitswissenschaften, Kaunas, Lithuania*, [2]*Universitätskliniken Kaunas, Plastic and Reconstructive Surgery, Kaunas, Lithuania*

**Background:**
Among Caucasians, basal cell carcinoma, classified as a non-melanoma type of skin cancer, stands as the most encountered form of cancer. A significant majority of these tumors (roughly 80-90%) manifest on facial regions that receive direct sunlight exposure. Within this subset, lesions located on the nose present the most formidable challenge for surgeons, chiefly due to anatomical intricacies, functional considerations, and aesthetic concerns.
The objective of the study was to assess the dispersion of tumor localization and size, morphological classifications, and the results of surgical intervention, encompassing the degree of radicality attained. Additionally, the study sought to scrutinize the correlation between the level of radical excision and the subsequent recurrence rate.

**Materials and methods:**
The study utilized information sourced from the Health Information Centre at the Hygiene Institute. Permissions to implement this study were granted by the Department of Bioethics, LUHSH (BEC – MF – 123. 16–12–2022). The authors conducted a retrospective investigation, scrutinizing 343 instances of nasal skin cancer over a four-year timeframe, starting from January 1, 2019, to December 31, 2022. Medical records were selected based on ICD – 10 – AM code: C44.3. Statistical analysis of the data was carried out using IBM SPSS Statistics 27.0 software.

**Results:**
The study encompassed patients with an average age of 75.18 years. The number of female participants notably exceeded that of males (252 females and 91 males), a difference found to be statistically significant ($p < 0.001$, $\chi 2 = 75.571$). The most prevalent tumor localizations were the left side of the nose, accounting for 87 cases (25.4%), and the dorsum nasi, accounting for 85 cases (24.8%). On average, tumor sizes were 0.79 ± 0.38 cm in females and 0.82 ± 0.44 cm in males. The infiltrative morphological type was the most frequently observed, with 243 cases (70.8%). In terms of tumor removal, excision with fasciocutaneous plastic surgery was significantly more prevalent, constituting 262 cases (76.4%) out of the total ($p < 0.001$, $\chi 2 = 306.9$). Complete excision was achieved in 271 lesions (79.0%), while incomplete excision occurred in 58 lesions (16.9%). Notably, there was a substantial correlation between non – radical excisions and a higher recurrence rate compared to radical excisions (24.1% and 6.3% respectively) ($p < 0.001$, $\chi 2 = 18.108$). For non – radically excised tumors, further management more frequently involved re – excision within 0 – 6 months, accounting for 23 cases (39.7%), and re – excision upon recurrence, accounting for 17 cases (29.3%).

**Conclusion:**
Nasal reconstruction entails the application of a diverse set of surgical techniques that must be intricately tailored to account for variables such as tumor localization, size, depth, and patient-specific requirements. Despite the scrupulous adherence of surgeons to visual guidelines for excising basal cell carcinomas, the incidence of non – radical procedures cannot be entirely obviated. Nonetheless, we are confronted with the challenge posed by the escalating incidence of BCC cases, a consequence of demographic aging patterns. It is judicious to consider the incorporation of the Mohs micrographic technique as a means to enhance surgical outcomes and elevate the degree of radicality.
